# Microscopy

**Published:** 1873-12

**Authors:** S. P. Cutler

**Affiliations:** Chairman, Memphis, Tenn.


### ARTICLE III.

### *Report of the Committee on Microscopy.*

BY S. P. CUTLER, M. D., D. D. S., CHAIRMAN, MEMPHIS, TENN.

Read before the Tennessee Dental Association, October, 1873.

Microscopy of a left lower molar of a stout, healthy farmer, bilious temperament, 36 years of age. This tooth has some unusual descriptive and microscopic features connected with its history. This tooth is well formed aud developed, the points of the fangs are completely ossified, they naturally coming in contact; this ossification belongs to the cementum only, not involving the dentine. The tooth has a cavity on ante. prox. aspect, not extending to pulp cavity, though near it; had been troublesome for some time.

On extraction, (which was a difficult process,) there came away with the tooth all the bone included between the fangs, as the fangs could not yield. On inspection I found this included bone quite firmly attached to fangs.

I made a microscopic section by sawing through the centre longitudinally up to the enamel, including fragment of bone. On holding this prepared specimen to the light, and looking through, it presents on one side or one fang in places not continuous, that peculiar ligaline or horny appearance so often witnesesd in teeth of old persons, and sometimes in younger: hence, diminished vital resistance and ossific encroachments in consequence follow.

Under the microscope this specimen has some unusual features not seen before by your committee. There are normal tubuli and occluded, or nearly so, tubuli in the hyaline structure ; also secondary dentine to a considerable extent, the pulp cavity being greatly narrowed, presenting the tortuous irregular tubular structure so often witnessed in the class of teeth above named.

The included fragment of alveolus presents the most unusual and remarkable features ever witnessed by your committee ; in some places this fragment of bone has the normal microscopic structures, *i. e.*, *lacunæ* and *canaliculi*, in other places the *lacunæ* and *canaliculi* present that appearance pertaining to extreme age, an obliteration to a great extent of *canaliculi* and inclosing of the *lacunæ*. In other regions, there are none of the microscopic bone structure, but instead there are the original cartillaginous stores of cells not having been differentiated in the slightest dégree towards bone structures ; there are the nice shaped smooth and perfect cartilage cells just the same as witnessed in all true original bone cartilage and in other cartilage, which vary somewhat. This portion of the fragment of bone appears to be just as firm and hard as the balance of the bone ; it also has the usual Haversian canals through it. There is no cementasis of any portion of the fang as might have been expected in a tooth with so many peculiarities.

The question in this connection is, how is it possible for cartilage to become hardened with lime salts without the normal bone structures; and how the lime salts could work their way through the cartilaginous structure so as to completely harden the bone, though not properly ossified, as that term means true bone formation ? The process must have been osmosis, as there are no apparent communications from one cartilage cell to another cell.

This may not be new to all, though it is to your committee. May we not expect to find still other anomalies in our special department.

---

